# A Systematic Review of Global Drivers of Ant Elevational Diversity

**DOI:** 10.1371/journal.pone.0155404

**Published:** 2016-05-13

**Authors:** Tim Szewczyk, Christy M. McCain

**Affiliations:** 1 Department of Ecology & Evolutionary Biology, University of Colorado, Boulder, Colorado, United States of America; 2 University of Colorado Museum of Natural History, University of Colorado, Boulder, Colorado, United States of America; Field Museum of Natural History, UNITED STATES

## Abstract

Ant diversity shows a variety of patterns across elevational gradients, though the patterns and drivers have not been evaluated comprehensively. In this systematic review and reanalysis, we use published data on ant elevational diversity to detail the observed patterns and to test the predictions and interactions of four major diversity hypotheses: thermal energy, the mid-domain effect, area, and the elevational climate model. Of sixty-seven published datasets from the literature, only those with standardized, comprehensive sampling were used. Datasets included both local and regional ant diversity and spanned 80° in latitude across six biogeographical provinces. We used a combination of simulations, linear regressions, and non-parametric statistics to test multiple quantitative predictions of each hypothesis. We used an environmentally and geometrically constrained model as well as multiple regression to test their interactions. Ant diversity showed three distinct patterns across elevations: most common were hump-shaped mid-elevation peaks in diversity, followed by low-elevation plateaus and monotonic decreases in the number of ant species. The elevational climate model, which proposes that temperature and precipitation jointly drive diversity, and area were partially supported as independent drivers. Thermal energy and the mid-domain effect were not supported as primary drivers of ant diversity globally. The interaction models supported the influence of multiple drivers, though not a consistent set. In contrast to many vertebrate taxa, global ant elevational diversity patterns appear more complex, with the best environmental model contingent on precipitation levels. Differences in ecology and natural history among taxa may be crucial to the processes influencing broad-scale diversity patterns.

## Introduction

Over the last two decades, a resurgence of interest in the large-scale patterns and drivers of species diversity has shown that elevational diversity is quite variable within and among taxa [1–4]. Myriad hypotheses have been proposed to explain the variation in diversity observed across latitudinal and elevational gradients [5–7]. Global analyses of the diversity of vertebrate and plant taxa across elevational gradients suggest some combination of the taxon's biology [4,8,9], geometric constraints [10], and the current climate [11,12] as the most likely drivers. Ants, like other taxa, show a variety of elevational diversity patterns globally [13–16], though neither the patterns nor the underlying drivers have been evaluated comprehensively across replicated gradients.

Elevational gradients provide compact, globally replicated systems for assessing the relative support for hypothesized diversity drivers [1,7,17]. Because different mountain ranges vary in characteristics such as climate and area distribution, comparisons of mountain ranges can decouple the variables and constraints that are confounded along the latitudinal gradient [7]. Global elevational gradients thus provide a robust system for evaluating diversity patterns and drivers [7,12].

Most research effort has been on vertebrate and plant taxa, though the majority of animal species are insects [18–21]. Ants in particular are ecologically diverse, relatively well-described, and have a wide variety of impacts as competitors, predators, scavengers, and seed-dispersers among others [22,23]. Unlike most insect taxa, the ants used in species-level identifications are wingless workers. Their restricted mobility heavily reduces the impact of windblown accidentals in estimating a species' elevational range since workers are unlikely to become airborne. Rather, long-range dispersal requires a queen who must then found a colony and rear the first brood to produce the workers that are typically collected for identification. Workers are consequently unlikely to be detected at elevations where their species cannot persist at least through a season. A range of factors have been implicated as drivers of ant diversity along elevational gradients by various studies, although no comprehensive analysis of single factors or the complexity of their interactions across all suitably-sampled datasets exists to date.

### Hypotheses

#### Thermal energy

Thermal energy has seen support as a driver of ant diversity [24,25] with several proposed mechanisms. Warmer temperatures may allow longer foraging periods [22] or increased food resources through increased productivity [26]. Alternatively, the metabolic theory of ecology (MTE) posits that metabolic rates drive ecological and evolutionary processes. Metabolic rates and chemical reactions increase with temperature, so speciation may increase correspondingly [27,28]. Regardless of mechanism, temperature-based hypotheses all predict a close relationship between temperature and diversity across the elevational gradient [5,26,27]. Because temperature declines on average 6°C for each 1000m gained in elevation [29], these hypotheses predict a corresponding monotonic decline in diversity from the mountain base to the summit.

#### Mid-domain effect

The mid-domain effect (MDE) is a null model based on the geometric constraints imposed by a bounded spatial domain [30,31]. It predicts the pattern of diversity that would be expected if these geometric constraints were the only factor affecting the distribution of species’ ranges. For example, if species ranges were placed randomly on an island with the only condition being that each range is wholly contained on the island, diversity would tend to be higher in the interior and decline toward the island boundaries. On elevational gradients, the simulated random placement of observed elevational ranges between the mountain base and summit results in highest diversity at the middle elevations with symmetric declines toward the low- and high-elevational boundaries [31–33]. In simulations using the empirical range size distribution, the expected mean range size at each elevation can be predicted across the elevational gradient [34]. If ant elevational diversity is driven by the MDE, such simulations should predict empirical diversity and mean range size across the gradient [34].

#### Area

The geographical area hypothesis, based on the species-area relationship, predicts that as the area in an elevational band increases, diversity in that band should increase [35,36]. Typically, this relationship is linear on a log-log scale [10,37,38]. Over broad spatial scales, larger areas allow for larger ranges, decreasing extinction probability and increasing speciation probability through the introduction of a barrier ([37] and references therein). Over narrow spatial scales, larger areas will likely include more habitats, increasing the probability of detecting additional species from adjacent habitats [35,37,39]. However, the effect of area may be greater over the larger spatial scales described by regional studies since sampling area is standardized across elevations in local studies [38,40].

#### Elevational climate model

The elevational climate model (ECM) approximates productivity and proposes that the combination of temperature and precipitation drives diversity, predicting highest diversity at the warmest, wettest elevations [12]. The diversity pattern predicted by the ECM consequently depends on the local mountain climate. On arid mountains, water availability is typically highest at middle elevations due to the dry climate at the base and increased runoff toward the summit [2,41]. Thus, water limitation restricts diversity toward the base while temperature restricts diversity toward the summit, resulting in a mid-elevation diversity peak. On mountains in wet climates water is plentiful, so temperature drives diversity, resulting in highest diversity at the base and declining diversity toward the summit [12]. The ECM offers testable predictions where fine-scale, reliable global productivity data are lacking.

We employ a set of selection criteria for datasets used in the main analyses [4,9,42]. These criteria, each of which targets a source of bias, restrict the datasets used to those that capture the underlying diversity pattern across the elevational gradient as a whole with reasonable accuracy. While datasets not meeting our criteria can be used to address other questions, for our purposes they unfortunately do not reliably contribute the appropriate, unbiased information.

Our aim is a synthetic understanding of what environmental factors, independently and simultaneously, are key drivers of ant diversity. We reanalyze published studies of montane ant diversity, assessing the impact of sampling completeness and bias, such as undersampling at low elevations, elevationally-biased sampling, and large-scale deforestation effects [11,17,42]. With appropriately-sampled ant datasets, we evaluate various predictions of diversity theory, including energetic, mid-domain effect, area, climate hypotheses, and their interactions.

## Materials and Methods

### Data

**Fig 1:**
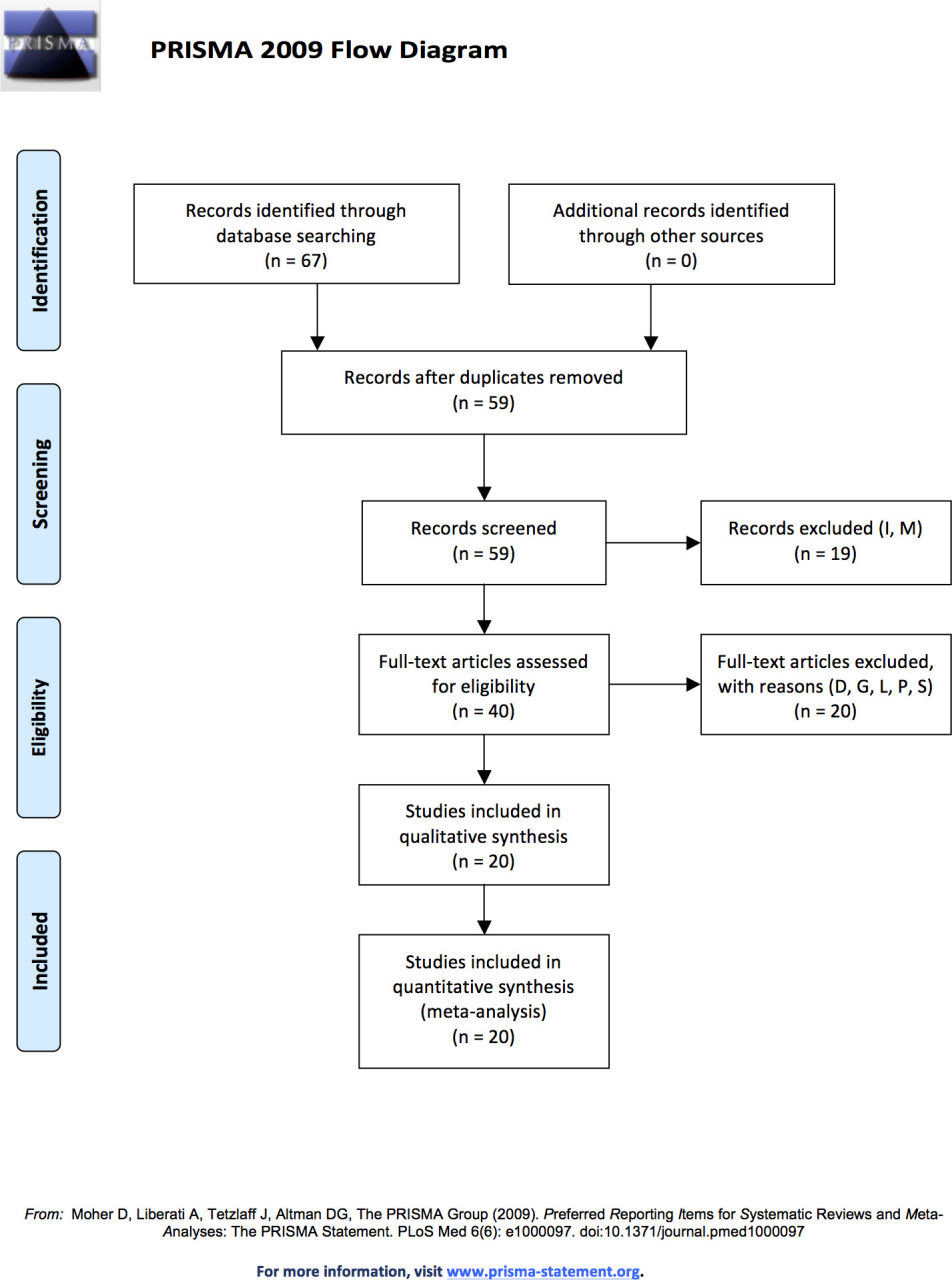
PRISMA flow diagram. Flow diagram showing the selection process for studies on ant elevational diversity. Database searches returned 67 possible data sources. Several studies used previously published data, leaving 59 unique datasets. Of those, 19 were excluded due to either insufficient elevational diversity data (I) or just a scattering of sampling sites spread across multiple gradients (M). The remaining 40 were evaluated using the *a priori* criteria (see text), with 20 excluded due to heavy disturbance (D), elevational sampling gaps >500m (G), lack of sampling within the lowest 400m (L), sampling of less than 70% of the gradient (P), elevationally biased or minimal sampling (S), or some combination. See additional transect details in S2 Table and additional PRISMA details in S2 Text.

To identify local-scale datasets of ant diversity sampled along elevational transects, we conducted literature searches (2014) using combinations of 'ant', 'elevation(-al)', 'altitud(-inal)', 'gradient', 'diversity', 'richness’, and 'insect' as keywords using Web of Knowledge and Google Scholar. To locate regional-scale datasets of ant diversity, we searched for publications with 'ants of' in the title; this search returned guides to the ant fauna of a geopolitical region, typically compiled from museum records and data collected over many years, detailing each recorded occurrence of each ant species in that region. Sixty-seven datasets were identified as possible sources for ant diversity across elevations (Fig 1; S1 and S2 Tables; S2 Text). In two cases, the authors sampled both sides of a mountain range with no shared sample sites, so the two transects were treated as separate transects [15,43]. Two datasets described just a scattering of sites spread across multiple gradients (M) and 17 provided insufficient elevational diversity data (I). For example, some regional datasets did not include elevations for many localities or for many species. Some local datasets sampled only 2–3 sites along a gradient or included abundances but not diversity. See S1 Text for additional details. Forty datasets were unique and provided data on ant diversity for a single gradient or region.

**Fig 2:**
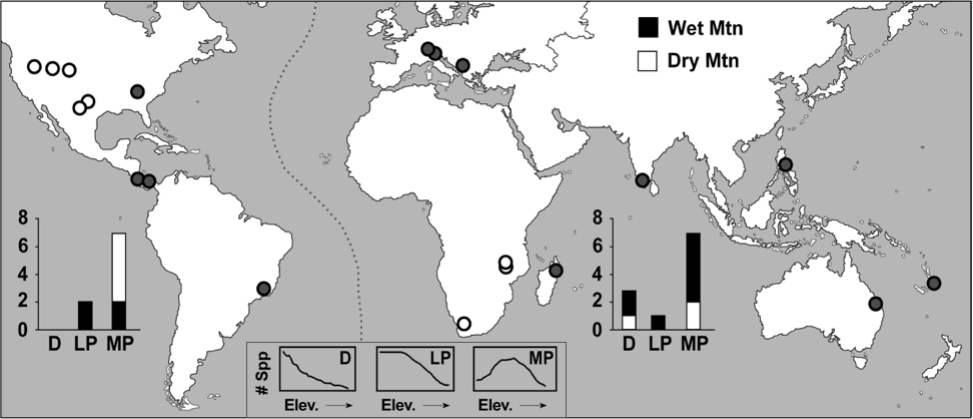
Map of ant elevational diversity datasets (*n* = 20). The distribution of ant study sites (circles), the three main elevational richness patterns for the eastern (*n* = 11) and western (*n* = 9) hemispheres (bars), and the number of patterns on wet and dry based mountains (black & white). (D = decreasing, LP = low plateau, MP = mid-elevational peak; see embedded figures and text for definitions).

To identify datasets robust for a comparative analysis of elevational diversity, we required that a study describe ant diversity along an elevational gradient or within a mountainous region and meet five *a priori* criteria: (1) high sampling effort with standardized methods across elevations; (2) sampling of ≥ 70% of the elevational gradient; (3) sampling within the lowest 400m of the gradient; (4) no sampling gaps > 500m in elevation; and (5) relatively little anthropogenic disturbance (e.g., widespread deforestation). These criteria were adapted from previous studies (e.g., [4,9]) and are necessary to ensure an accurate description of the naturally occurring pattern [11,17,18,42,44,45]. While these criteria do reduce the sample size, they are essential for this type of analysis. For example, because diversity patterns differ primarily across the lower portion of the mountain and all common patterns show a decline from intermediate elevations to the summit (Fig 2; S1 Fig), reliable estimates of the diversity across the lower half of the gradient is essential. Datasets violating the third criterion, consequently, may not contribute constructive information and are in fact biased toward showing a declining trend. Similarly, an overall pattern cannot be confidently discerned with large elevational gaps in sampling. However, violations of the second criterion were allowed when the unsampled region was primarily at high elevations where declining diversity had been sufficiently demonstrated. See S1 Text for further details.

Twenty datasets met the *a priori* sampling criteria (Fig 2), representing six biogeographical provinces in both temperate and tropical regions, and consisting of eleven local studies and nine regional studies. Additionally, eight studies were in arid climates and twelve were in wet climates. This number of suitable well-sampled datasets is similar to some harder to survey vertebrate taxa, like bats (e.g., 22 bat datasets: [12]), and comparable proportions of appropriately sampled studies (20/40 = 50%) have been found for birds (41%: [4]), bats (44%: [12]), and vertebrate ectotherms (51%: [46]), with higher proportions for reptiles (67%: [9]) and small non-volant mammals (73%: [11]). Of the ant datasets that were excluded due to sampling issues, 60% either did not sample >70% of the gradient or did not sample the lowest 400m (S2 Table). Because diversity typically decreases beyond a certain elevation, lack of sampling across the lower portions of an elevational gradient creates a bias toward detecting decreases in diversity (panel ‘b’ in S1 Fig; S2 Table), regardless of the underlying pattern [42,45]. Similarly, minimal sampling at the mountain base may bias detection toward mid-elevation peaks in diversity. Such biases preclude the robust determination of the underlying pattern. The use of rigorous *a priori* standards is crucial to understanding and disentangling the drivers of diversity.

Elevational ranges of ant species in each study were interpolated. That is, a species was assumed present at all elevations between the lowest and highest observed elevations. Although interpolation may artificially inflate the reported diversity at middle elevations [47], the majority of species in these datasets were detected at all sampled elevational bands within their elevational range. Thus, the impact of interpolation was minimal and did not alter any overall diversity patterns. We therefore employed interpolation uniformly to account for potential undersampling and for standardization among datasets. Each elevational gradient was divided into 100m bands (i.e., 0−99m, 100−199m, etc.), and diversity was estimated as the number of species' ranges in each band.

Sampling methods varied among studies, though most used mini-Winkler traps, pitfall traps, or both (S1 Table). If authors reported data from both standardized and non-standardized methods (e.g., haphazard hand collection), only the former was included in this reanalysis. In some cases, authors reported diversity based on rarefaction methods. While rarefaction better accounts for rare or difficult to detect species and may allow more accurate comparisons [48–50], many studies did not report these values and did not provide sufficient information for their calculation. Additionally, rarefied diversity does not allow for hypothesis testing using species ranges (i.e., MDE and EGCM). Therefore, we used only interpolated diversity of sampled species.

Ant elevational diversity was classified into five broad patterns using previous definitions (S1 Fig)[4,9]. Decreasing patterns show highest diversity in the lowest elevational band with diversity declining as elevation increases. Low plateaus have consistently high diversity across at least the lowest 300m followed by a monotonic decline in diversity. Mid-elevation peaks have the highest diversity at middle elevations (> 300m from the base) and with 25% greater diversity than at the base. Increasing patterns have increasing diversity with increasing elevation. Lastly, no pattern was detected when none of these definitions was met. More patterns are possible [4], but the observed ant patterns were characterized by these five classifications and the latter two only for datasets that did not meet our sampling criteria.

For area analyses, digital elevation models (DEMs) were downloaded from CGIAR-CSI (srtm.csi.cgiar.org). These rasters are derived from the USGS/NASA SRTM data at ~90m x 90m resolution with a vertical error of < 16m. Using ArcGIS, rasters were converted into an Albers Equal Area projection centered on each study site. For regional studies, the boundary of the study area was the corresponding geopolitical region. For local studies, the study area boundary was determined by using mountain ridges and major watersheds to isolate the focal mountain within a 30km buffer around the sampling locations [38]. Alternative delineation methods and buffer sizes did not qualitatively alter area profiles. To estimate the area within each 100m elevational band, we calculated the area of the hypotenuse plane of each raster cell and then, within each 100m band, summed the calculated areas of all cells.

Rasters of climate data at 1km x 1km resolution were downloaded from WorldClim (worldclim.org) and converted into Albers Equal Area projections as above. Only mean annual temperature and annual precipitation were used because of high collinearity among other variables. Though the resolution is relatively coarse, the quality was consistent across the globe. Additionally, finer resolution would likely have little qualitative effect because the climatic variables were averaged within each 100m band in a study area. The actual climatic conditions experienced by the ants depend on local conditions, including variables such as slope, aspect, and vegetation structure. However, many studies sampled across multiple habitats at each elevation, so an average is frequently more appropriate. In the absence of climate measurements at the point of collection, metrics like mean annual temperature serve as a reasonable approximation. Studies were classified into two broad climate categories based on the climate of the mountain base (arid: humidity index < 0.5, wet: humidity index > 0.5; [51]).

### Hypothesis Tests

#### Thermal energy

If thermal energy is the primary driver of ant diversity, then diversity should decline monotonically on each elevational gradient, mirroring the declining temperature. Though ants experience variable microclimates at any elevation, such microhabitat temperature is variability around the general decline of temperature with increasing elevation at the scales considered in these analyses. Decreasing patterns should consequently be most common regardless of other mountain characteristics such as precipitation or area. We evaluated three predictions based on the thermal energy hypothesis. First, simple linear regressions were used to test for a positive, linear relationship between mean annual temperature and diversity for each study. Additionally, two predictions of the metabolic theory of ecology (MTE) were tested: (1) a linear relationship according to the equation ln(S) = b*(kT)^*-1*^ *+ c*, where *S* is the diversity within an elevational band, *k* is Boltzmann's constant (*k = 8*.*62x10*^*-5*^ eV*K^*-1*^), and *T* is the annual mean temperature in Kelvin, and (2) a slope of -0.7 < *b* < -0.6 [28,46,52]. To test the MTE predictions, we combined all ant datasets and used a simple linear regression [46,52]. All temperature analyses were repeated using area-standardized diversity (below).

#### Mid-domain effect

To test the MDE predictions, we randomized the placement of elevational ranges using the observed range size distribution within the spatial boundaries of each elevational gradient [53]. We used the empirical range size distributions because the distribution of elevational range sizes drives the steepness of the expected hump-shaped diversity curve [33]. Four studies did not provide the elevational range of each species and were not included in tests of the MDE predictions (S1 Table). In 100,000 simulations for each study, we calculated predicted diversity as the number of ranges occurring in each elevational band and mean range size as the average size of those ranges. The predictive ability of the MDE was assessed in four ways. We performed simple linear regressions of the mean MDE-predicted diversity values with the observed diversity values. We also calculated the proportion of observed diversity values falling within the middle 95% of the simulated diversity values. We repeated both of these analyses using mean range size at each elevation in place of diversity. The MDE may only be evident when diversity is standardized for area at each elevation [11,38]. This was tested only with simple linear regressions of area-standardized diversity (detailed below) and mean predicted diversity values because area-standardized diversity values are not on a natural scale, precluding direct comparisons of magnitude.

#### Area

We evaluated four predictions of the area hypothesis. First, to test the strength of the species-area relationship, we used simple linear regressions after log-transforming diversity and area [10,37,54]. Second, the area hypothesis predicts that standardizing diversity for elevational area should alter the diversity pattern [38]. Third, standardizing diversity for area should also alter the elevation of the diversity peak [38]. To standardize for area, we first estimated an overall *z* for montane ants using *S = cA*^*z*^ with all studies combined. This method reduces bias from extreme *z* values occasionally observed on mountains [38], though varying *z* beyond the 95% confidence limits had little qualitative effect on the resulting patterns. Using this averaged *z*, we calculated area-standardized diversity for each elevation in each study as *S/A*^*z*^, where *S* and *A* are the interpolated diversity and area for a given elevational band. The area-standardized diversity patterns were then characterized using the descriptions above. A paired t-test was used to determine whether area-standardization significantly altered the elevation of the diversity peak. Fourth, to test whether the species-area relationship was stronger in regional studies than in local studies [40], we used a Mann-Whitney U-test to compare coefficients of determination.

#### Elevational climate model (ECM)

We tested four predictions of the ECM. First, to test the prediction that mid-peaks are more common on arid than wet mountains, we used a Fisher's Exact Test to detect if wet and arid climates were positively associated with high diversity at the base (decreasing and low plateaus) and low diversity at the base (mid-peaks) respectively. Second, to test whether temperature predicts diversity better on wet than arid mountains, we used simple linear regressions and compared the coefficients of determination with a Mann-Whitney U-test. Third, to evaluate whether diversity at the mountain base is higher on wet than arid mountains, we compared the proportion of diversity at the base (S_base_/S_gradient_) using a Mann-Whitney U-test. Using proportions allows for better comparison of the diversity pattern shape and accounts for large differences in total diversity across studies. Fourth, to test whether precipitation limits diversity at the base of arid mountains, we correlated base precipitation with the proportion of diversity at the base using Spearman's Rho.

#### Interactions: environmentally & geometrically constrained model (EGCM) and multiple regression

We used two methods to assess the effects of each hypothesized driver simultaneously: an environmentally and geometrically constrained model (EGCM) and multiple regression. We used an EGCM [55] to incorporate MDE constraints in addition to the other potential drivers. The EGCM framework relies on the prediction that the geometric constraints imposed by a bounded domain (i.e., the MDE) affect large-ranged species more than small-ranged species. Small-ranged species therefore better reflect the influence of the environmental gradient in the absence of any mid-domain effects. We explored range-size cutoffs of 1/4, 1/5, 1/6, and 1/7 of the mountain gradient to define the small-ranged species group. Excluding elevations near the boundaries where mid-domain effects are strongest [55], we used the diversity of small-ranged species at each cutoff to fit an environmental model with the log diversity of each elevational band predicted by log area, mean annual temperature, and annual precipitation, using AIC to determine the optimal model for each cutoff. We used the predictions from the optimal environmental models as probability distributions for 5,000 simulations, randomly placing the elevational midpoints of the large-ranged species along the corresponding gradient. This method for placing large-ranged species is identical to the method for the MDE, but with the placement of their range mid-points biased by the environmental model predictions rather than being drawn from a uniform distribution. We selected the optimal cutoff based on the strength of the fit between these simulations and the observed large-ranged species diversity for each transect [55]. The EGCM analysis used the same subset of datasets used to evaluate the MDE. For the multiple linear regression, we used the same model structure as the environmental component of the EGCM, but fit the models using all species rather than just the small-ranged species. We determined the optimal model with AIC.

We assessed specific predictions of each hypothesis rather than performing formal meta-analyses. Meta-analyses of effect sizes use weights based on the strength of the results, ideally representing the sampling effort. However, standard metrics of sampling effort, such as sample size, do not translate well for these elevational studies. In this case, the sample size in each study is the number of elevational bands. Thus, the sample size reflects the height of the mountain rather than the actual sampling effort. Additionally, studies used a variety of sampling techniques (S1 Table) and included compilations based on museum records, rendering a universal estimate of sampling effort somewhat discretionary. Conducting a formal meta-analysis using arbitrary weights may lead to erroneous results [45,56] and was consequently avoided here.

## Results

Appropriately sampled ant diversity datasets showed three of the patterns detailed above. Most transects showed mid-elevation peaks, while monotonic declines and low plateaus occurred in equal proportion (Fig 2; panel ‘a’ in S1 Fig). The distribution of patterns was nearly identical between local and regional scales: both showed primarily mid-peaks with equal numbers of low plateau and decreasing patterns (panel ‘a’ in S1 Fig; S1 Table).

The excluded datasets showed five patterns (panel ‘b’ in S1 Fig). Mid-peaks were most common as in the well-sampled gradients, though a substantial number of decreasing patterns were detected. These were observed largely in studies that did not sample within the lowest 400m. Low plateaus, increases, and no pattern occurred in equal, low numbers (panel ‘b’ in S1 Fig; S2 Table).

### Hypothesis Tests

#### Thermal energy

**Fig 3:**
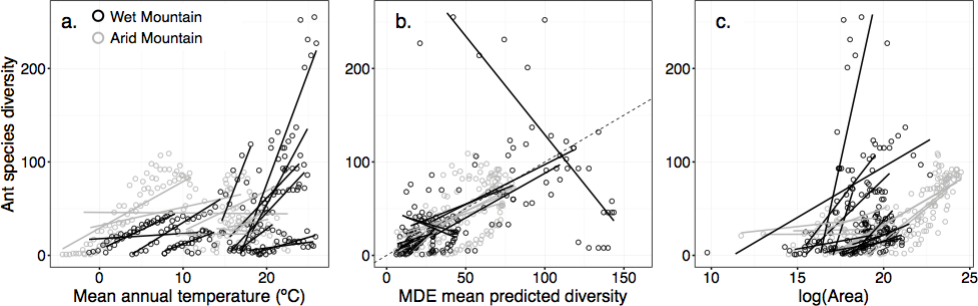
Scatterplots with regression lines for temperature, area, and MDE mean predictions with observed diversity showing wet and area mountains. Points represent elevational sites (black: wet mountains; gray: arid mountains) and lines are the regression lines for each study. Panels show the ant diversity predicted by (a) mean annual temperature, (b) mid-domain effect mean predicted diversity, and (c) log-transformed area. The dotted line in (b) represents the 1:1 relationship that would be expected with a perfect MDE fit.

**Fig 4:**
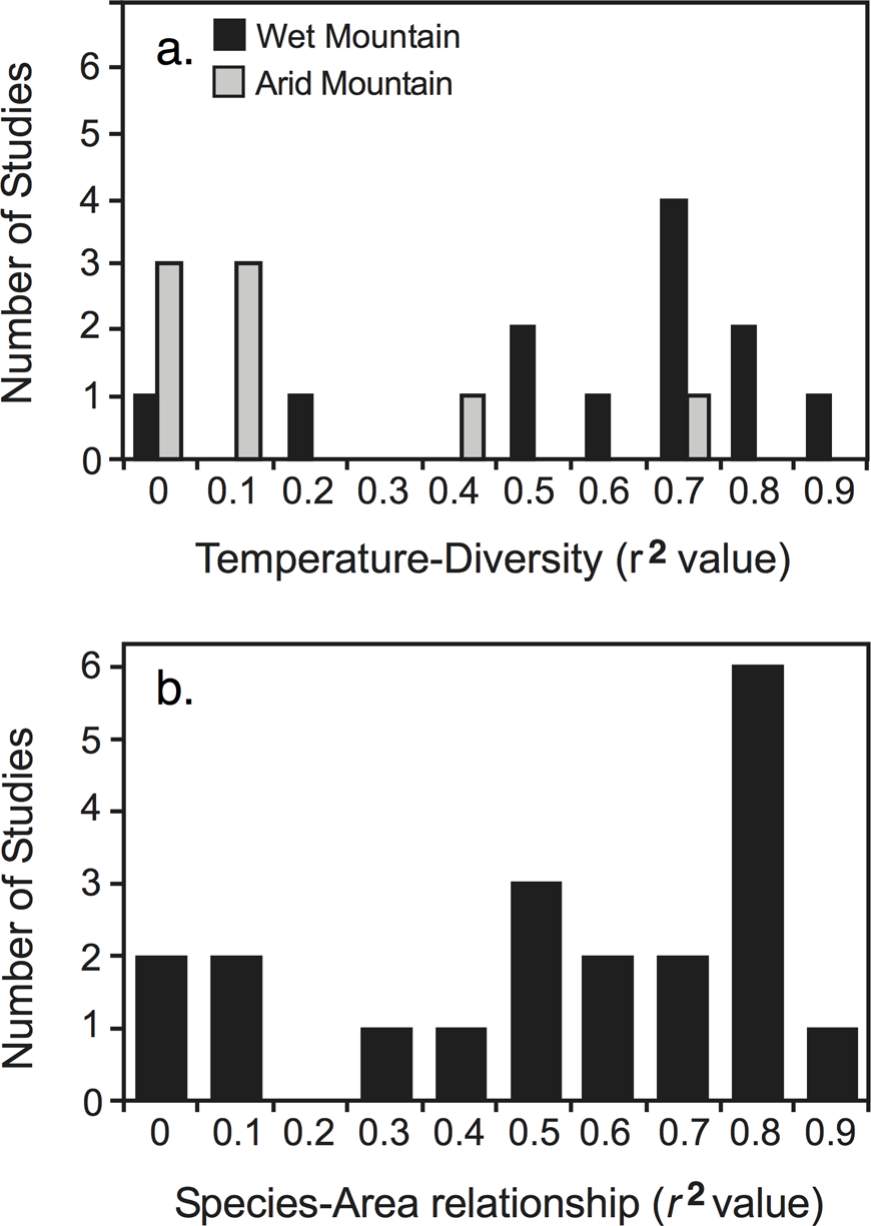
Regression analyses of temperature-diversity and area-diversity relationships in well-sampled ant datasets. (a) Fits to the temperature-ant diversity relationship (*n* = 20) with mean *r*^*2*^ = 0.465 ± 0.076 (SE). Wet mountains showed a significantly better fit than did arid mountains (*r*^*2*^_*wet*_ = 0.635 ± 0.078, *n* = 12; *r*^*2*^_*arid*_ = 0.210 ± 0.095, *n* = 8; *P* = 0.003). (b) Fits to the area-ant diversity relationship (*n* = 20) with mean *r*^*2*^ = 0.585 ± 0.068 (SE).

Thermal energy was not well supported as an independent driver of ant diversity. Only 15% of studies detected a monotonic decrease with increasing elevation. No area-standardized patterns showed monotonic decreases. Though a positive, significant relationship with temperature was detected in 60% of studies, the *r*^*2*^ distribution was bimodal with many studies showing a very poor fit (Fig 3A; Fig 4A; *r*^*2*^ mean = 0.465, median = 0.550) reflecting a poor ability to predict diversity based on temperature. With area-standardization, 70% of studies showed a positive, significant relationship, though fits were similarly poor (*r*^*2*^ mean = 0.462, median = 0.458). Additionally, the individual relationships were predominantly curvilinear rather than the linear relationships predicted by a strong temperature driver. In support of the first prediction of the MTE, a linear relationship between log-transformed diversity and the inverse of temperature fit the data better than a curvilinear relationship (delta AIC = 12.06), but the slope did not fall within the predicted bounds (*P* < 0.001, 95% CI: -0.487 < *β* < -0.258, predicted: -0.7 < *β* < -0.6). Area-standardized diversity showed similar results (P < 0.001, 95% CI: -0.384 < *β* < -0.198).

#### MDE

**Fig 5:**
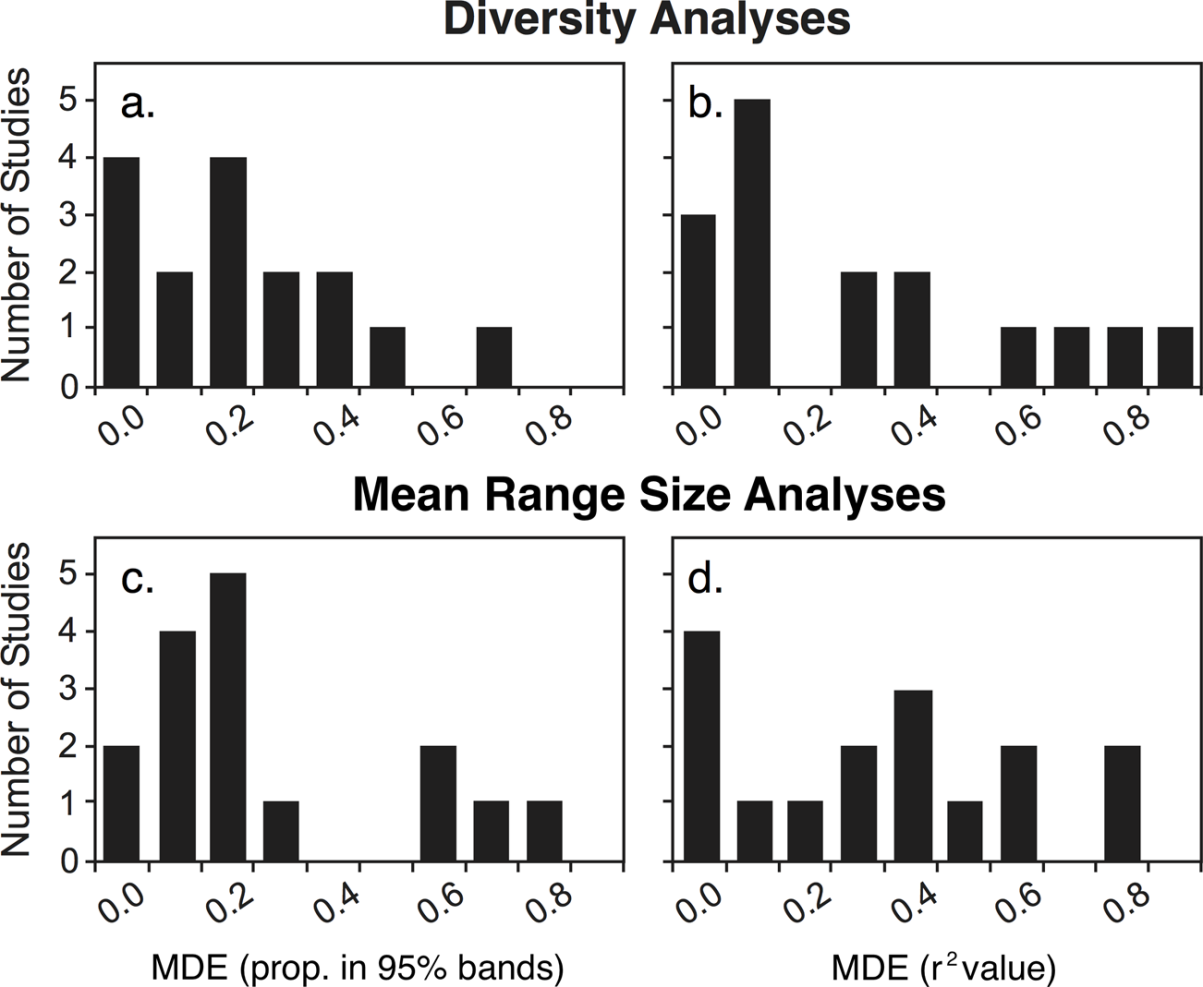
Evaluation of mid-domain effect (MDE) predictions (*n* = 16). MDE simulations poorly predicted diversity both in (a) the proportion of observed values falling within the 95% predictive bands (mean = 0.257 ± 0.044 (SE)) and in (b) the *r*^*2*^ values from linear regressions of the MDE-predicted means and observed diversity values (mean *r*^*2*^ = 0.354 ± 0.067). Mean range size was poorly predicted by the MDE simulations measured both by (c) the mean range sizes falling within the 95% predictive bands (mean = 0.335 ± 0.055) and by (d) linear regressions of the MDE-predicted means and observed mean range size values (mean *r*^*2*^ = 0.384 ± 0.059).

There was little support for the MDE as an independent driver. The MDE generally predicted diversity poorly with either the proportional or linear model method (Fig 3B; Fig 5A and 5B; proportion of points within 95% bands: mean = 0.257, median = 0.218; linear models: *r*^*2*^ mean = 0.354, median = 0.274). Standardizing for area slightly improved the linear model fit (*r*^*2*^ mean = 0.396, median = 0.318). The MDE predicted mean range size somewhat better than it did diversity, but overall fits were still generally low (Fig 5C and 5D; proportion within 95% bands: mean = 0.335, median = 0.278; linear models: *r*^*2*^ mean = 0.384, median = 0.387).

#### Area

There was mixed support for the area hypothesis. A positive, significant relationship between area and diversity was seen in 80% of studies (Fig 3C; Fig 4B; *r*^*2*^ mean = 0.585, median = 0.685). With area standardization, the diversity pattern changed in 50% of studies and the diversity peak shifted significantly upward (*P* = 0.01, *t*_*19*_ = 2.77), though only 35% of studies showed a shift > 300m. The effect of area did not differ with scale (*P* = 0.82, *W* = 46).

#### ECM

There was mixed support for the ECM. Mid-peaks were not more likely on arid mountains (Fig 2; *P* = 0.32, *ω* = 4.63). However, temperature predicted diversity significantly better on wet mountains (Fig 4A; *P* = 0.003, *W* = 85), and a significant relationship between temperature and diversity was detected on 83% of wet mountains and 25% of arid mountains. The proportion of diversity at the base was significantly higher on wet mountains (*P* = 0.025, *W* = 19). There was no significant correlation on arid mountains between the proportion of diversity at the base and the precipitation at the base (*P* = 0.096).

#### Interactions

**Fig 6:**
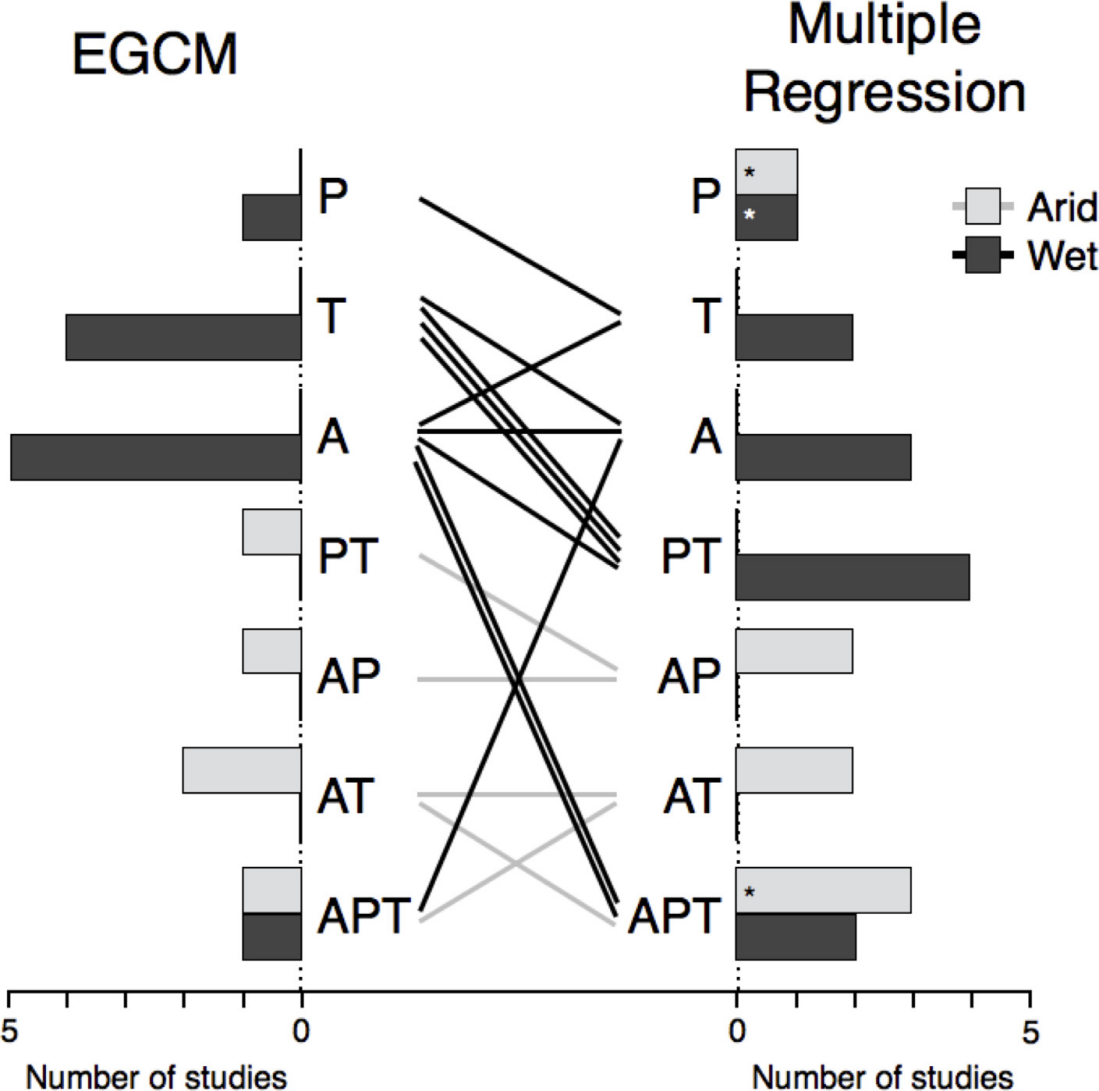
Optimal environmental models for the EGCM (*n* = 16) and the multiple regression (*n* = 20). The EGCM models were fit using only small-ranged species in each transect while the multiple regression used the diversity of all species. In the full model, log diversity in each elevational band was predicted by log area (A), mean annual temperature (T), and annual precipitation (P). Generally, wet mountain ant diversity (dark gray) was best predicted by one environmental variable, most often either area or temperature. In contrast, arid mountain ant diversity (light gray) was best predicted by models that included two or three variables, most often with area or temperature as one of the variables. Along most transects (lines), the optimal model differed between the EGCM and the multiple regression. Bars with transects included in the multiple regression but not in the EGCM are marked with (*).

In the EGCM, small-ranged species diversity was predicted reasonably well by the best environmental models (*r*^*2*^ mean = 0.661, median = 0.723, range = 0.189–0.982). The large-ranged species diversity pattern was very well predicted by the EGCM simulations in most studies, though the fit was rather poor in two studies (*r*^*2*^ mean = 0.843, median = 0.957, range = 0.197–0.988). Additionally, inclusion of geometric constraints improved fit in 62.5% of studies, though there was no significant improvement of fit (*P* = 0.093). Each possible environmental model (e.g.: ‘temp’; ‘temp + area’; ‘precip + area’; etc.) was the optimal model for at least one study (Fig 6, left bars). With the exception of one dataset, the optimal models for all wet-based mountains included just one environmental variable. In contrast, the optimal models for all arid-based mountains included multiple variables. The multiple regression fit was comparable to that of the EGCM (*r*^*2*^ mean = 0.830, median = 0.923, range = 0.041–0.997). The optimal regression model was different than the optimal EGCM model for all but three studies (Fig 6, lines). Each possible model was the optimal regression model for at least one study, with a similar distinction in model complexity between wet- and arid-based mountains, though ‘precip + temp’ was the best regression model on many wet gradients (Fig 6, right bars).

## Discussion

None of the four broad drivers assessed individually were universally supported in these ant diversity datasets. Rather, evaluation of interactions showed support for the influence of multiple drivers, including temperature, precipitation, and area, though the supported combination varied among mountain ranges. The supported drivers seem to be largely contingent on whether the mountain base is wet or arid, as shown in both the EGCM and the multiple regressions.

Additional factors may affect patterns of ant diversity, although they were not evaluated here due to lack of data availability across all gradients. Habitat complexity, vegetation structure, and leaf litter depth have been suggested in several systems [57–60]. Complications arise, however, both in identifying and quantifying the relevant habitat features [17], particularly in analyses spanning the globe. Biotic interactions have long been speculated to affect patterns of diversity [7], though the extent to which local interactions among individuals affect large scale patterns of diversity remains unclear [61,62]. Detailed data on potential competitors along each elevational gradient, paired with sufficient knowledge of species-specific interactions, are required to assess this hypothesis. Finally, evolutionary history may impact diversity patterns [63]; rigorous evaluation relies on the development of species-level, time-calibrated phylogenies. However, evolutionary models based on range sizes and niche spaces predict consistent elevational diversity patterns within a region and primarily mid-elevation peaks in diversity [4,64], rather than the variation observed. Additionally, past climatic fluctuations may have resulted in lowland biotic attrition which would affect the observed elevational diversity patterns [65]. During interglacial periods, species would be expected to shift upward in elevation due to warmer temperatures. At the lowest elevations, no species would exist to replace those that moved uphill, depressing the diversity at low elevations and resulting in low plateaus or mid-peaks [65,66]. Finally, these analyses are of course observational rather than experimental, limiting the scope of mechanistic inferences.

Temperature generally does not predict ant diversity well across these elevational gradients regardless of area standardization. Further, we failed to find convincing evidence for the MTE or any simple relationship with thermal energy. This is contrary to expectations based on the ectothermic physiology of ants and their thermophilic foraging behavior (e.g., [13,14,24,25,67,68]). While local ant diversity may be consistent with one formulation of the MTE along a latitudinal gradient [69], the controversial hypothesis has not seen broad support [46,70]. There are several reasons that ant elevational diversity may not show a linear relationship with temperature. Ants behaviorally moderate the temperatures they experience through nest site and architecture [71] and by altering temporal patterns of foraging [22] to minimize exposure to extreme temperatures, potentially obfuscating the effects of temperature. The temperatures experienced by many ants would be more closely approximated by soil temperature, which could be affected by elevational changes in the vegetation structure, though soil temperatures still generally decline with elevation. Further, these individual-level effects of temperature may not scale up directly or linearly to communities of ants spread along an entire elevational gradient.

Perhaps more likely is that the effect of temperature is contingent upon water availability. Energy and water have been implicated as important drivers of diversity and abundance in both plants and animals [3,26,72], with direct physiological effects and indirect effects through productivity and food resources [4,6,12,26,72]. Previous work has shown that local ant community diversity is affected by temperature both directly and indirectly through productivity [69], suggesting that, when comparing globally distributed elevational gradients, the relationships may be complex. Indeed, while ant diversity across elevations was largely consistent with the predictions of the ECM, the effects of temperature and precipitation appear to be more nuanced.

Area likewise was not consistently supported, though it predicted diversity well along the majority of transects and likely plays a role in shaping elevational patterns of ant diversity. Several regional datasets included here are remarkably well-described by geometric drivers [73]. However, several other regional datasets showed very poor fits. We detected no difference between regional and local gradients contrary to predictions. This emphasizes the importance of comprehensive, global analyses. Further it suggests that local studies along elevational gradients may occur at intermediate scales for ants, resulting in similar dynamics in local and regional studies of elevational diversity rather than in clear differences among the spatial resolutions [17]. Alternatively, the local species diversity may be influenced by regional area indirectly via the regional species pool [39,74].

In contrast to area, no support was found for the MDE, whether assessed independently or with area-standardized diversity. The MDE has had a highly contentious history with highly variable predictive ability across multiple taxa [4,11,73,75,76]. Studies evaluating elevational gradients globally, however, have generally found poor support for the MDE [4,9,12,19,33]. The MDE is not useful for predicting ant elevational diversity.

Diversity hypotheses are not mutually exclusive, of course, and likely act in tandem to shape the patterns observed across elevational gradients [33,55,63]. The EGCM and multiple regression both predicted diversity quite well along most gradients, with no significant difference with the inclusion of geometric constraints in the EGCM. Along nearly all gradients, the optimal environmental model differed between the EGCM, fit with just small-ranged species, and the multiple regression, fit with all species (Fig 6). Thus, while incorporating geometric effects did not improve the overall predictive power, the differences between the EGCM and the multiple regression suggest that large- and small-ranged species are responding differently. The EGCM assumes that the environmental drivers of large- and small-ranged species are identical, attributing any differences to the impacts of geometric constraints, which theoretically and empirically have more influence on large-ranged species [33,55]. Given the broader ecological tolerances of large-ranged species compared to elevationally-restricted species, the validity of this assumption is arguable. Additionally, the EGCM method assumes that the observed elevational ranges accurately reflect each species’ environmental tolerance. However, the elevational ranges of rare or patchily distributed species are more likely to be underestimated, potentially biasing which species are used in fitting the environmental model. Consequently, the disparity in optimal models between the EGCM and the multiple regression has two possible interpretations. All species along a transect may be driven by the same environmental variables, captured by the EGCM by removing any effects of geometric constraints on broadly distributed species. The multiple regression, by ignoring geometric constraints, would not accurately estimate the effects of the environmental variables. Alternatively, environmental effects may vary among species. The multiple regression effectively marginalizes across all species to estimate the overall response. Under this interpretation, the intentionally biased subset used by the EGCM would fail to estimate the true impacts of each environmental driver.

Regardless, both the EGCM and the multiple regression indicate a dichotomy based on the precipitation levels at the mountain base. That is, the distribution of optimal models is sharply divided between wet and arid climates. The EGCM shows that one dominant factor drives ant diversity on most wet-based gradients. This was most often either area or temperature. In contrast, the best environmental models for arid climates always included multiple variables. The multiple regression similarly shows diversity in arid climates as generally driven by a combination of factors, typically including area. This suggests a broad, underlying influence of precipitation with the diversity pattern further modified by area, temperature, or a combination depending on the gradient.

Ant diversity is similar to that of other taxa in that a variety of elevational patterns is seen globally. The diversity patterns of vertebrate taxa investigated so far, however, seem to be driven by a more cohesive combination of factors. The elevational diversity patterns of birds, bats, and small non-volant mammals are all likely shaped by both temperature and precipitation [4,11,12,38] despite each taxon showing a different overall distribution of diversity patterns. Reptile diversity is more closely tied to temperature, though precipitation and area seem to play a role as well [9,46]. Although ants, as ectotherms, would intuitively be affected largely by temperature, the robust patterns of ant diversity across elevational gradients instead suggest a complex interplay of multiple drivers.

In conclusion, ant elevational diversity is shaped by several factors, with the current precipitation regime altering both their number and identity. Ant gradients, in conjunction with those of other taxa, suggest that a taxon’s ecology and natural history may be critical to the processes influencing broad-scale patterns. The rigorous analysis of more insect and invertebrate taxa at a global extent in addition to a focus on gathering key data to test other potential drivers (e.g., biotic interactions and natural history) will provide the perspective required to understand how diversity is distributed and how to better predict future changes.

## Supporting Information

S1 Fig Pattern distribution for included and excluded datasets. (a) Included datasets (*n* = 20) most often showed highest ant diversity at intermediate elevations, though both decreasing and low plateau patterns occurred. Local and regional datasets did not differ in the pattern distribution. (b) Excluded datasets (*n* = 20; reasons for exclusion = D, G, L, P, S in Fig 1 & S2 Table) were more varied, with mid-peaks, decreasing patterns, low plateaus, increasing patterns, and no pattern. Most decreasing patterns were reported in studies that did not sample within the lowest 400m of the gradient.
(PDF)

S1 Table Details and references of ant elevational gradients used in analyses. (PDF)

S2 Table Details and references of ant datasets excluded from analyses. (PDF)

S1 Text Methods supplement. Expanded description of dataset selection process.
(PDF)

S2 Text PRISMA checklist. (PDF)
