# Supplementary material for: A Systematic Review of Global Drivers of Ant Elevational Diversity
Source: PLoS One. 2016 May 13;11(5):e0155404. doi: 10.1371/journal.pone.0155404 (PMC4866765; doi:10.1371/journal.pone.0155404)

S4 Figure. Pattern distribution for included and excluded datasets. (a) Included datasets (*n*=20) most often showed highest ant diversity at intermediate elevations, though both decreasing and low plateau patterns occurred. Local and regional datasets did not differ in the pattern distribution. (b) Excluded datasets (*n*=20; reasons for exclusion = D, G, L, P, S in Fig. 1 & S2 Table) were more varied, with mid-peaks, decreasing patterns, low plateaus, increasing patterns, and no pattern. Most decreasing patterns were reported in studies that did not sample within the lowest 400m of the gradient.

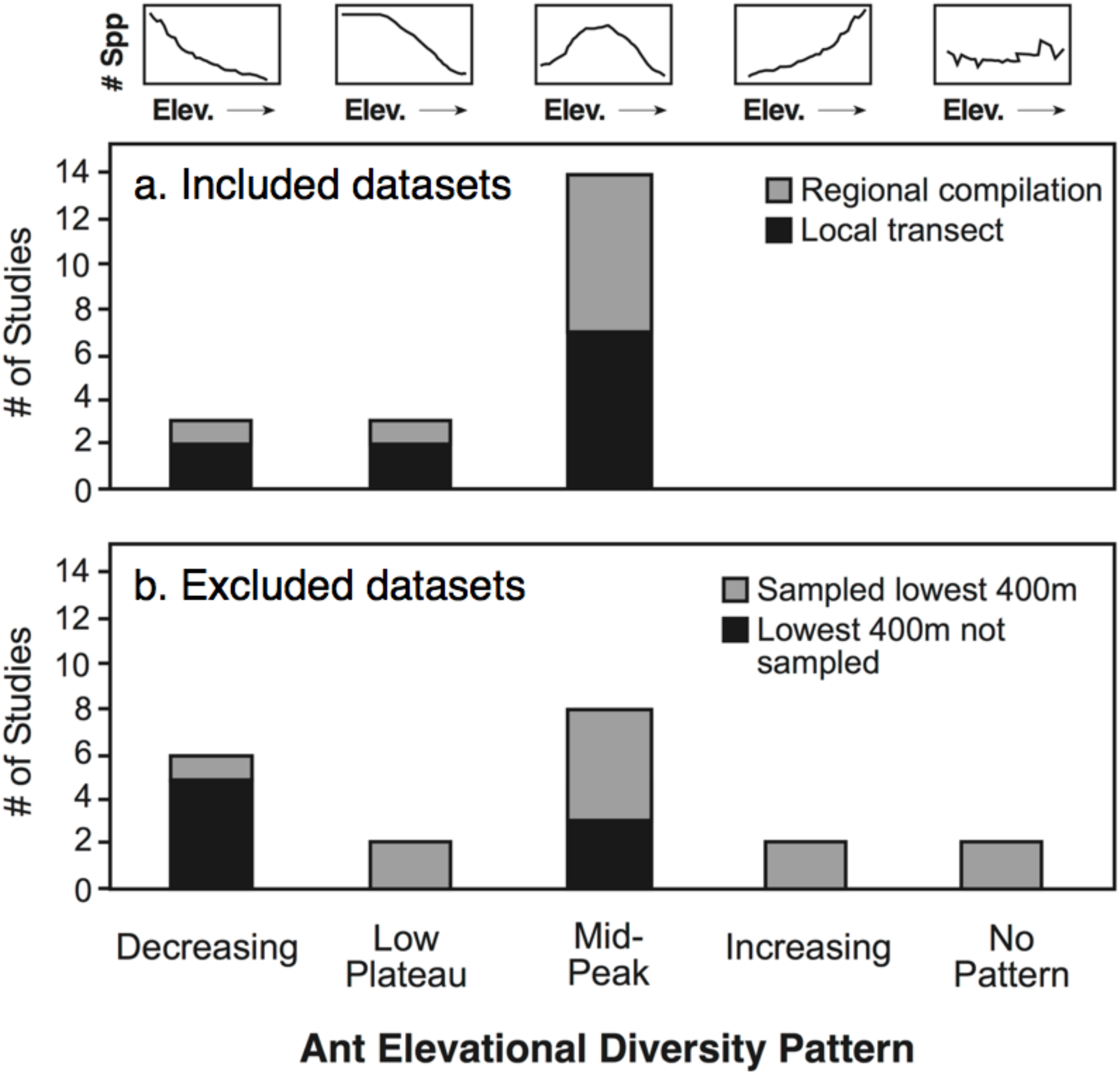

Supplement: S1 Fig — (a) Included datasets (n = 20) most often showed highest ant diversity at intermediate elevations, though both decreasing and low plateau patterns occurred. Local and regional datasets did not differ in the pattern distribution. (b) Excluded datasets (n = 20; reasons for exclusion = D, G, L, P, S in Fig 1 & S2 Table) were more varied, with mid-peaks, decreasing patterns, low plateaus, increasing patterns, and no pattern. Most decreasing patterns were reported in studies that did not sample within the lowest 400m of the gradient. (PDF) [file pone.0155404.s001.pdf]
