# Supplementary material for: A Systematic Review of Global Drivers of Ant Elevational Diversity
Source: PLoS One. 2016 May 13;11(5):e0155404. doi: 10.1371/journal.pone.0155404 (PMC4866765; doi:10.1371/journal.pone.0155404)
Supplement: S1 Table — (PDF) [file pone.0155404.s002.pdf]

S1 Table. Details and references of ant elevational gradients used in analyses. List of robust and informative elevational gradients in ant species richness. Each denoted by study site (reference), diversity pattern (D: decreasing; LP: low-plateau; MP: mid-peak), data scale (L: local; R: regional), survey methods (B: baited traps, F: canopy or bark fogging; H: hand collection; L: litter sifting; M: malaise traps; P: pitfall traps; R: museum records), latitude, mountain base climate, % of gradient sampled, meters unsampled at mountain base, mountain height (m), temperature r^2 values, MDE diversity and range r^2 values, species-area r^2 values, best fit EGCM environmental model, EGCM environmental model r^2 values, EGCM simulation r^2 values, and total number of species and genera on gradient.

Study Site (Reference)	Div. Pattern	Scale	Survey Methods	Lat.	Climate	% Grad.	Unsamp. base (m)	Mtn Hgt (m)	Temp *r2*	MDE Div. *r2*	MDE Rng *r2*	Area *r2*	EGCM best mod	EGCM Env. *r2*	EGCM Sim. *r2*	Species (Genera)
Soutpansberg, South Africa (Munyai & Foord 2012)	D	L	P	-23.0	Arid	72.3	106	938	0.745	---	---	0.887	---	---	---	78 (28)
Utah, USA (Allred 1982)	MP	R	H	39.5	Arid	78.9	52	3459	0.149	0.111	0.080	0.509	ATP	0.698	0.969	121 (23)
Colorado, USA (Gregg 1963)	MP	R	H	39.0	Arid	95.1	49	3367	0.492	0.162	0.027	0.809	AT	0.326	0.978	149 (40)
Nevada, USA (Wheeler & Wheeler 1986)	MP	R	H, R	38.5	Arid	92.8	9	3835	0.000	0.896	0.174	0.900	AT	0.981	0.975	157 (28)
W. Texas, USA**	MP	R	B, H, L, R	32.8	Arid	92.1	0	2498	0.148	0.379	0.353	0.907	AP	0.981	0.975	149 (35)
Chisos Mtns, TX, USA (Van Pelt 1983)	MP	R	B, H, L, R	29.3	Arid	76.2	348	1706	0.007	0.406	0.417	0.002	TP	0.855	0.968	81 (29)
Soutpansberg, South Africa (Munyai & Foord 2012)	MP	L	P	-23.0	Arid	83.4	142	1067	0.141	---	---	0.563	---	---	---	78 (28)
Cederburg Wilderness, South Africa (Botes et al. 2006)	MP	L	P	-32.4	Arid	97.8	5	1964	0.000	---	---	0.004	---	---	---	85 (24)
Montenegro (Karaman 2011)	D	R	H	42.3	Wet	77.5	100	2194	0.928	0.070	0.455	0.767	T	0.794	0.283	87 (28)
McPherson Range, Australia (Burwell & Nakamura 2011)	D	L	F, H, L	-28.2	Wet	66.9*	300	1196	0.749	0.651	0.556	0.806	A	0.777	0.970	169 (57)
Voralberg, Austria (Glaser 2006)	LP	R	R	47.3	Wet	59.6*	12	2853	0.851	0.405	0.618	0.110	T	0.909	0.197	65 (17)
Volcan Barva, Costa Rica (Longino & Colwell 2011)	LP	L	L	10.3	Wet	67.3*	50	2897	0.826	0.711	0.880	0.487	P	0.719	0.682	401 (70)
Espinhaco, Brazil (Araujo & Fernandes 2003)	LP	L	B, H	-19.4	Wet	56.4*	322	1241	0.775	0.950	0.406	0.773	T	0.982	0.961	39 (15)
South Tirol, Italy (Hellrigl 2003)	MP	R	H, R	46.8	Wet	62.6*	9	3674	0.029	0.068	0.203	0.194	T	0.189	0.922	70 (24)
Smoky Mtns, TN, USA (Lessard et al. 2007)	MP	L	L, M, P	35.6	Wet	75.4	161	1807	0.701	0.102	0.625	0.845	ATP	0.727	0.953	38 (20)
Mt Isarog, Philippines (Samson et al 1997)	MP	L	P, H	13.7	Wet	75.6	250	1983	0.593	0.190	0.368	0.373	A	0.575	0.971	52 (20)
Western Ghats, India (Sabu et al. 2008)	MP	L	L	11.7	Wet	58.1*	300	2322	0.205	0.026	0.811	0.543	A	0.200	0.845	29 (18)
Bocas del Toro, Panama (Olson 1994)	MP	L	L, P	8.8	Wet	58.1*	300	2375	0.799	---	---	0.686	---	---	---	196 (-)
Ambohitsitondroina, Madagascar (Fisher 1998)	MP	L	L, P	-15.1	Wet	95.5	25	2053	0.506	0.177	0.067	0.684	A	0.349	0.988	272 (28)
Fiji (Sarnat & Economo 2012)	MP	R	ALL	-17.7	Wet	98.1	0	1324	0.654	0.359	0.096	0.850	A	0.579	0.914	174 (42)

* < 70% of gradient sampled, but primarily upper elevations where diversity had already decreased monotonically
** Moody & Francke 1982; Cockendolpher & Francke 1990

S1: References

1. Allred DM. Ants of Utah. Gt Basin Nat. 1982;42: 415–511.

2. Araújo LLM, Fernandes GWG. Altitudinal patterns in a tropical ant assemblage and variation in species richness between habitats. Lundiana. 2003;4: 103–109.

3. Botes A, McGeoch MA, Robertson HG, van Niekerk A, Davids HP, Chown SL. Ants, altitude and change in the northern Cape Floristic Region. J Biogeogr. 2006;33: 71–90. doi:10.1111/j.1365-2699.2005.01336.x

4. Burwell CJ, Nakamura A. Distribution of ant species along an altitudinal transect in continuous rainforest in subtropical Queensland, Australia. Mem Queensl Museum. 2011;55: 391–412.

5. Cokendolpher JC, Francke OF. The ants (Hymenoptera, Formicidae) of western Texas. Part II. Subfamilies Ecitoninae, Ponerinae, Pseudomyrmecinae, Dolichoderinae, and Formicinae. Spec Publ Mus Tex Tech Univ. Lubbock, TX: Texas Tech Press; 1990.

6. Fisher BL. Ant diversity patterns along an elevational gradient in the Réserve Spéciale d'Anjanaharibe-Sud and on the western Masoala Peninsula, Madagascar. Fieldiana Zool. 1998;90: 39–67.

7. Glaser F. Biogeography, diversity, and vertical distribution of ants (Hymenoptera: Formicidae) in Vorarlberg, Austria. Myrmecological News. 2006;8: 263–270.

8. Gregg RE. The Ants of Colorado. Univ. of Colorado Press. Boulder, Colorado, xvi. Denver, CO: University of Colorado Press; 1963.

9. Hellrigl K. Faunistik der Ameisen und Wildbienen Südtirols (Hymenoptera: Formicidae et Apoidea). Gredleriana. 2003;3: 143–208.

10. Karaman MG. Zoogeography, diversity and altitudinal distribution of ants (Hymenoptera: Formicidae) in the Mediterranean and the oro-Mediterranean parts of Montenegro. North West J Zool. 2011;7: 26–34.

11. Lessard JJP, Dunn RRR, Parker CCR, Sanders NNJ. Rarity and diversity in forest ant assemblages of Great Smoky Mountains National Park. Southeast Nat. 2007;0: 215–228.

12. Longino JT, Colwell RK. Density compensation, species composition, and richness of ants on a neotropical elevational gradient. Ecosphere. 2011;2: art29. doi:10.1890/ES10-00200.1

13. Moody JV, Francke OF. The ants (Hymenoptera, Formicidae) of western Texas Part I. Subfamily Myrmicinae. Lubbock, TX: Texas Tech Press; 1982.

14. Munyai TC, Foord SH. Ants on a mountain: spatial, environmental and habitat associations along an altitudinal transect in a centre of endemism. J Insect Conserv. 2012;16: 677–695. doi:10.1007/s10841-011-9449-9

15. Olson DM. The distribution of leaf litter invertebrates along a neotropical altitudinal gradient. J Trop Ecol. 1994;10: 129–150.

16. Sabu TK, Vineesh P, Vinod K. Diversity of forest litter-inhabiting ants along elevations in the Wayanad region of the Western Ghats. J Insect Sci. 2008;8: 1–14.

17. Samson DA, Rickart EA, Gonzales PC. Ant diversity and abundance along an elevational gradient in the Phillippines. Biotropica. 1997;29: 349–363.

18. Sarnat E, Economo E. The ants of Fiji. Berkeley and Los Angeles, CA: UC Publications in Entomology, University of California Press; 2012.

19. Van Pelt A. Southwestern Association of Naturalists Ants of the Chisos Mountains, Texas (Hymenoptera: Formicidae). Southwest Nat. 1983;28: 137–142.

20. Wheeler G, Wheeler J. The ants of Nevada. Los Angeles, CA: Natural History Museum of Los Angeles County; 1986.
