# Supplementary material for: A Systematic Review of Global Drivers of Ant Elevational Diversity
Source: PLoS One. 2016 May 13;11(5):e0155404. doi: 10.1371/journal.pone.0155404 (PMC4866765; doi:10.1371/journal.pone.0155404)
Supplement: S2 Table — (PDF) [file pone.0155404.s003.pdf]

**S2 Table. Details and references of ant datasets excluded from analyses.** Datasets that were not used in analyses, including the study site (reference), the reported diversity pattern (D: decreasing; I: Increasing; LP: low-plateau; MP: mid-peak, and No Pattern), the data scale (local or regional), and the reason(s) the study was not included (D: large portion of gradient had anthropogenic disturbance; G: sampling gaps between sites were >500m; I: insufficient or no elevational diversity data; L: no sampling in the lowest 400m; M: multiple gradients or sites too far apart; P: <70% of elevational gradient sampled and not primarily upper elevaitons where diversity had already decreased monotonically; R: repeated data or non-independent transect; S: sampling effort minimal or biased elevationally).

| Study Site (Reference) | Reported Pattern | Scale | Exclusion Reason |
|---|---|---|---|
| Alps, Tirol, Austria (Kofler 1978) | --- | Regional | I |
| Andes, Colombia (van der Hammen & Ward 2005) | MP | Local | L, S |
| Andes, Lambayeque, Peru (Delgado et al. 2008) | D | Local | L, P, S |
| Andes, Venezuela (Janzen 1976) | LP | Local | G |
| Andohahela, Madagascar (Fisher 1999) | MP | Local | P |
| Andringitra, Madagascar (Fisher 1996) | D | Local | L, P |
| Appalachian Mtns, NC, VA, MD; USA (McCoy 1990) | --- | Local | I |
| Appalachian Mtns, VA, USA (Wang et al. 2001) | D | Local | I |
| Cederburg Wilderness, South Africa (Botes et al. 2006) | MP | Local | L |
| Chiricahua Mtns, AZ, USA (Anderson 1997) | MP | Local | S |
| Colorado, USA (Sanders 2002) | MP | Regional | R |
| Coastal Cordillera, Venezuela (Lattke & Riera-Valera 2012) | --- | Local | I, L, P |
| Costa Rica (Janzen 1973) | --- | Local | I, G |
| Eastern Australia (Majer et al. 2001) | D | Local | G, M |
| Georgia (Chaladze 2012) | MP | Regional | I |
| Georgia, USA (Ipser et al. 2004) | MP | Regional | S |
| Gunung Mulu, Sarawak (Collins 1980) | --- | Local | I |
| Himalayas, India (Bharti 2008) | --- | Regional | I, L |
| Himalayas, Jammu-Kashmir, India (Bharti & Sharma 2009) | --- | Local | I, G, L, P |
| Himalayas, Jammu-Kashmir, India (Bharti et al. 2013) | MP | Local | G |
| Imatong Mtns, Sudan (Weber 1943) | --- | Local | I |
| Kinabalu, Malaysia (Brühl et al. 1999) | D | Local | L, S |
| Kinabalu, Malaysia (Malsch et al. 2008) | D | Local | L |
| Maloti-Drakensberg, South Africa (Bishop et al. 2014) | MP | Local | I |
| Manongarivo, Madagascar (Fisher 2002) | LP | Local | S |
| Minorca, Spain (Guillem 2009) | --- | Local | I, P |
| Monts Doudou, Gabon (Fisher 2004) | No Pattern | Local | D, S |
| Nevada, USA (Sanders 2002) | MP | Regional | R |
| Northeastern USA (Del Toro 2013) | --- | Local | M |
| Okinawa, Japan (Ito et al. 1998) | --- | Local | I, P, S |
| Shoshone Mtns, NV, USA (MontBlanc et al. 2007) | MP | Local | L, P |
| Sierra Nevada de Santa Marta, Colombia (Guerrero & Sarmiento 2010) | D | Local | L, P |
| Smoky Mtns, TN, USA (Cole 1940) | LP | Regional | R |
| Smoky Mtns, TN, USA (Geraghty et al. 2007) | D | Local | R |

| Study Site (Reference) | Reported Pattern | Scale | Reason for Exclusion |
|---|---|---|---|
| Smoky Mtns, TN, USA (Sanders et al. 2007) | D | Local | R |
| Smoky Mtns, TN, USA (Whittaker 1952) | --- | Local | I |
| Smoky Mtns, TN, USA (Zelikova et al. 2008) | D | Local | I, R |
| South Korea (Kwon et al. 2014) | D | Local | S |
| South Sinai, Egypt (Orabi et al. 2011) | No Pattern | Local | G |
| South Tirol, Italy (Glaser 2008) | --- | Regional | I |
| Spring Mtns, NV, USA (Sanders 2003) | I | Local | P |
| Spring Mtns, NV, USA (Sanders 2003) | I | Local | P |
| Spring Mtns, NV, USA (Sanders 2003) | MP | Local | P |
| Tatra Mtns, Poland (Woyciechowski 1993) | --- | Local | I, L, P |
| Utah, USA (Sanders 2002) | MP | Regional | R |
| Volcan Barva, Costa Rica (Atkin & Proctor 1988) | --- | Local | I |
| Volcan Barva, Costa Rica (Colwell et al. 2008) | LP | Local | R |

## S2: References


1. Andersen AN. Functional groups and patterns of organization in North American ant communities: a comparison with Australia. J Biogeogr. 1997;24: 433–460.
2. Atkin L, Proctor J. Invertebrates in the litter and soil on Volcan Barva, Costa Rica. J Trop Ecol. 1988;4: 307–310.
3. Bharti H. Altitudinal diversity of ants in Himalayan regions (Hymenoptera: Formicidae). Sociobiology. 2008;52: 305–322.
4. Bharti H, Sharma YP. Diversity and abundance of ants along an elevational gradient in Jammu-Kashmir Himalaya-I. Halteres. 2009;1: 10–24.
5. Bharti H, Sharma YP, Bharti M, Pfeiffer M. Ant species richness, endemicity and functional groups, along an elevational gradient in the Himalayas. Asian Myrmecology. 2013;5: 79–101.
6. Bishop TR, Robertson MP, van Rensburg BJ, Parr CL. Elevation-diversity patterns through space and time: Ant communities of the Maloti-Drakensberg Mountains of southern Africa. Gillman L, editor. Journal of Biogeography. 24 Jun 2014: 1–13. doi:10.1111/jbi.12368
7. Brühl CA, Mohamed M, Linsenmair KE. Altitudinal distribution of leaf litter ants along a transect in primary forests on Mount Kinabalu, Sabah, Malaysia. J Trop Ecol. 1999;15: 265–277.
8. Castro Delgado S, Vergara Cobian C, Arellano Ugarte C. Distribucion de la riqueza, composicion taxonomica y grupos funcionales de hormigas del suelo a lo largo de un gradiente altitudinal en el Refugio de Vida Silvestre Laquipampa, Lambayeque - Peru. Ecol Apl. 2008;7: 89–103.
9. Chaladze G. Climate-based model of spatial pattern of the species richness of ants in Georgia. J Insect Conserv. 2012;16: 791–800. doi:10.1007/s10841-012-9464-5
10. Cole A. A guide to the ants of the Great Smoky Mountains National Park, Tennessee. Am Midl Nat. 1940;24: 1–88.
11. Collins N. The distribution of soil macrofauna on the west ridge of Gunung (Mount) Mulu, Sarawak. Oecologia. 1980;44: 263–275.
12. Colwell RK, Brehm G, Cardelús CL. Global warming, elevational range shifts, and lowland biotic attrition in the wet tropics. Science (80- ). 2008;322: 258–261.

13. Del Toro I. Diversity of Eastern North American ant communities along environmental gradients. PLoS One. 2013;8: e67973. doi:10.1371/journal.pone.0067973

14. Fisher BL. Ant diversity patterns along an elevational gradient in the Réserve Naturelle Intégrale d'Andringitra, Madagascar. Fieldiana Zool. 1996;85: 93–108.

15. Fisher BL. Ant diversity patterns along an elevational gradient in the Réserve Spéciale de Manongarivo, Madagascar. Boissiera. 2002;59: 311–328.

16. Fisher BLBBL. Diversity patterns of ants (Hymenoptera: Formicidae) along an elevational gradient on Monts Doudou in Southwestern Gabon. Calif Acad Sci Mem. 2004;28: 269–286.

17. Geraghty MJ, Dunn RR, Sanders NJ. Body size, colony size, and range size in ants (Hymenoptera: Formicidae): are patterns along elevational and latitudinal gradients consistent with Bergmann's rule. Myrmecological News. 2007;10: 51–58.

18. Glaser F. Die Ameisenfauna (Hymenoptera, Formicidae) des Schlerngebiets (Italien, Sudtirol). Gredleriana. 2008;8: 467–496.

19. Guerrero RJ, Sarmiento CE. Distribucion altitudinal de hormigas (Hymenoptera, Formicidae) en la vertiente noroccidental de la Sierra Nevada de Santa Marta (Colombia). Acta Zool Mex. 2010;26: 279–302.

20. Guillem R. A survey of the ants of Minorca (Hymenoptera: Formicidae) with two new species for the island: Hypoponera punctatissima (Roger, 1859) and Temnothorax algiricus (Forel, 1894). Boletín la Asoc Española Entomol. 2009;33: 447–460.

21. Ipser RM, Brinkman MA, Gardner WA, Peeler HB. A survey of ground-dwelling ants (Hymenoptera: Formicidae) in Georgia. Florida Entomol. 2004;87: 253–260.

22. Ito Y, Takamine H, Yamauchi K. Abundance and species diversity of ants in forests of Yanbaru, the Northern Part of Okinawa Honto with special reference to effects of undergrowth removal. Entomol Sci. 1998;1: 347–355.

23. Janzen D. Sweep samples of tropical foliage insects: Description of study sites , with data on species abundances and size distributions. Ecology. 1973;54: 659–686.

24. Janzen D, Ataroff M, Farinas M, Reyes S. Changes in the arthropod community along an elevational transect in the Venezuelan Andes. Biotropica. 1976;8: 193–203.

25. Kofler A. Faunistik der Ameisen (Insecta: Hymenoptera, Formicoidea) Osttirols (Tirol, Österreich). Ber nat-med Verein Innsbruck. 1978; 117–128.

26. Kwon T-S, Kim S-S, Chun JH. Pattern of ant diversity in Korea: An empirical test of Rapoport's altitudinal rule. Journal of Asia-Pacific Entomology. Elsevier B.V.; 2014. doi:10.1016/j.aspen.2013.12.006

27. Lapolla JS, Suman T, Sosa-Calvo J, Schultz TR. Leaf litter ant diversity in Guyana. Biodivers Conserv. 2007;16: 491–510. doi:10.1007/s10531-005-6229-4

28. Lattke JE, Riera-valera MA. Diversidad de hormigas (Hymenoptera: Formicidae) en la hojarasca y suelo de selvas nubladas de la Cordillera de la Costa, Venezuela. Metod en Ecol y Sist. 2012;7: 20–34.

29. Majer JD, Kitching RL, Heterick BE, Hurley K. North-south patterns within arboreal ant assemblages from rain forests in eastern Australia. Biotropica. 2001;33: 643–661.

30. Malsch A, Fiala B, Maschwitz U, Mohamed M, Nais J, Linsenmair K. An analysis of declining ant species richness with increasing elevation at Mount Kinabalu, Sabah, Borneo. Asian Myrmecology. 2008;2: 33–49.

31. McCoy ED. The distribution of insects along elevational gradients. Oikos. 1990;58: 313–322.

32. MontBlanc EM, Chambers JC, Brussard PF. Variation in ant populations with elevation, tree cover, and fire in a pinyon-juniper-dominated watershed. West North Am Nat. 2007;67: 469–491.

33. Orabi GM, Semida FM, Abdel-Dayem MS, Sharaf MR, Zalat SM. Diversity patterns of ants along an elevation gradient at St. Catherine Protectorate, South Sinai, Egypt. Zool Middle East. 2011;54: 101–112. doi:10.1080/09397140.2011.10648882

34. Reymond A, Purcell J, Cherix D, Guisan A, Pellissier L. Functional diversity decreases with temperature in high elevation ant fauna. Ecol Entomol. 2013;38: 364–373. doi:10.1111/een.12027

35. Robertson HG. Comparison of leaf litter ant communities in woodlands, lowland forests and montane forests of north-eastern Tanzania. Biodivers Conserv. 2002;11: 1637–1652.

36. Sanders NJ. Elevational gradients in ant species richness: area, geometry, and Rapoport's rule. Ecography (Cop). 2002;1: 25–32.

37. Sanders NJ, Lessard J-P, Fitzpatrick MC, Dunn RR. Temperature, but not productivity or geometry, predicts elevational diversity gradients in ants across spatial grains. Glob Ecol Biogeogr. 2007;16: 640–649. doi:10.1111/j.1466-8238.2007.00316.x

38. Sanders NJ, Moss J, Wagner D. Patterns of ant species richness along elevational gradients in an arid ecosystem. Glob Ecol Biogeogr. 2003;12: 93–102. doi:10.1046/j.1466-822X.2003.00324.x

39. van der Hammen T, Ward P. Ants from the Ecoandes expeditions: diversity and distribution. Stud Trop Andean Ecosyst. 2005;6.

40. Wang C, Strazanac JS, Butler L. Association between ants (Hymenoptera: Formicidae) and habitat characteristics in oak-dominated mixed forests. Environ Entomol. 2001;30: 842–848.

41. Weber NA. The ants of the Imatong Mountains, Anglo-Egyptian Sudan. Bull Museum Comp Zool. 1943;93: 263–389.

42. Whittaker RH. A study of summer foliage insect communities in the Great Smoky Mountains. Ecol Monogr. 1952;22: 1–44.

43. Woyciechowski M. Ants (Hymenoptera, Formicidae) of the glades in the Tatra Mts (the Carpathians). Tiscia. 1993;27: 17–22.

44. Zelikova TJ, Dunn RR, Sanders NJ. Variation in seed dispersal along an elevational gradient in Great Smoky Mountains National Park. Acta Oecologica. 2008;34: 155–162. doi:10.1016/j.actao.2008.05.002
