# Supplementary material for: A Systematic Review of Global Drivers of Ant Elevational Diversity
Source: PLoS One. 2016 May 13;11(5):e0155404. doi: 10.1371/journal.pone.0155404 (PMC4866765; doi:10.1371/journal.pone.0155404)
Supplement: S1 Text — Expanded description of dataset selection process. (PDF) [file pone.0155404.s004.pdf]

S3 Methods supplement

This methods supplement further describes the study selection process.

The literature search returned 67 potential datasets. Of those, 8 used data that had been previously published ('R' in S2 Table). These 59 datasets were then screened to determine if they provided data of ant diversity along an elevational gradient. Two datasets reported on a scattering of sites spread across multiple elevational gradients rather than sites along one gradient ('M' in S2 Table) and 17 were determined to have insufficient elevational data ('I' in S2 Table). These datasets lacked sufficient data to be considered reasonably complete datasets of ant elevational diversity. The regional datasets listed as 'I' did not provide the elevations of many localities or for many species. Local datasets were listed as 'I' for several reasons. Some reported ant abundances, but not diversity. Others only sampled 2-3 elevations, so no reasonable estimate of trends across the elevational gradient could be discerned. Some only covered a few hundred meters of the elevational gradient. For datasets listed as 'I' due to these latter two reasons, S2 Table also lists related violations of the *a priori* sampling criteria.

This left 40 unique datasets that reported ant diversity data along what could be reasonably considered a continuous elevational gradient or within a region. We developed criteria for inclusion prior to the literature search based on previous studies to ensure that the data used would reflect the underlying diversity reasonably accurately [1-8]. Specifically, we required that each dataset: had no heavy disturbance ('D' in S2 Table), had no elevational gaps in sampling >500m ('G' in S2 Table), sampled within the lowest 400m of the gradient ('L' in S2 Table), had sampling sites spanning >70% of the gradient ('P' in S2 Table), and sampled without elevational bias and with reasonably intensive effort ('S' in S2 Table). Many of the datasets violated multiple criteria, and this final screening step returned 20 datasets that were appropriately sampled for this analysis.

Our questions and analyses relate to the gradient as a whole and, consequently, necessitate a reliable estimate of ant diversity across the entire gradient. Each criterion targets a specific aspect that is essential to capturing the elevational pattern and minimizing bias. While datasets that violate the criteria can be used perfectly validly for other purposes, they simply do not offer constructive information for our questions. In fact, datasets with no sampling in the lower 400m are more likely to report a declining elevational diversity pattern and, consequently, including them in this analysis would introduce systematic bias. Therefore, we restricted the analysis to datasets that were determined with *a priori* criteria to be adequately sampled for this analysis.

References:

1. McCain CM. Elevational gradients in diversity of small mammals. Ecology. 2005;86: 366–372.
2. McCain CM. Global analysis of bird elevational diversity. Glob Ecol Biogeogr. 2009;18: 346–360. doi:10.1111/j.1466-8238.2008.00443.x
3. McCain CM, Grytnes JA. Elevational gradients in species richness. Encyclopedia of Life Sciences. 2010. pp. 1–10. doi:10.1002/9780470015902.a0022548
4. McCain CM. Global analysis of reptile elevational diversity. Glob Ecol Biogeogr. 2010;19: 541–553. doi:10.1111/j.1466-8238.2010.00528.x
5. McCoy ED. The distribution of insects along elevational gradients. Oikos. 1990;58: 313–322.
6. McCoy ED. The "veiled gradients" problem in ecology. Oikos. 2002;99: 189–192.
7. Nogués-Bravo D, Araújo MB, Romdal T, Rahbek C. Scale effects and human impact on the elevational species richness gradients. Nature. 2008;453: 216–219. doi:10.1038/nature06812
8. Rahbek C. The role of spatial scale and the perception of large-scale species-richness patterns. Ecol Lett. 2005;8: 224–239. doi:10.1111/j.1461-0248.2004.00701.x
